# Extracorporeal blood purification in patients with liver failure: Considerations for the low-and-middle income countries of Latin America

**DOI:** 10.3389/fneph.2023.938710

**Published:** 2023-01-31

**Authors:** Vladimir Barrera Villanueva, Daniel Alejandro Barrera Amorós, Eira Ingrid Castillo Echeverria, Luis F. Budar-Fernández, Omar Israel Salas Nolasco, Luis A. Juncos, Lilia Rizo-Topete

**Affiliations:** ^1^ Division of Nephrology, Instituto Mexicano del Seguro Social, Unidad Médica de Alta Especialidad 14, Universidad Veracruz, Veracruz, Mexico; ^2^ Nephrologist, Instituto Mexicano del Seguro Social, Hospital General de Zona 71, Veracruz, Mexico; ^3^ Nephrologist, Hospital Español de Veracruz, Veracruz, Mexico; ^4^ Division of Nephrology, Central Arkansas Veterans Healthcare System and the University of Arkansas for Medical Sciences, Little Rock, Arkansas, EUA, United States; ^5^ Division of Nephrology, Hospital Unniversitario “Dr. José Eleuterio Gonzalez”, Universidad Autónoma de Nuevo León (UANL), Nuevo León, Mexico; ^6^ Division Internal Medicine, Hospital Christus Muguerza Alta Especialidad, Universidad de Monterrey (UDEM), Monterrey, Nuevo Leon, Mexico

**Keywords:** extracorporeal liver support devices, acute liver failure, continuous renal replacement therapy, extrarenal, extracorporeal liver assist device, acute liver failure (ALF), extra-renal, CRRT - continuous renal replacement therapy

## Abstract

Severe liver failure is common in Low-and-Medium Income Countries (LMIC) and is associated with a high morbidity, mortality and represents an important burden to the healthcare system. In its most severe state, liver failure is a medical emergency, that requires supportive care until either the liver recovers or a liver transplant is performed. Frequently the patient requires intensive support until their liver recovers or they receive a liver transplant. Extracorporeal blood purification techniques can be employed as a strategy for bridging to transplantation or recovery. The most common type of extracorporeal support provided to these patients is kidney replacement therapy (KRT), as acute kidney injury is very common in these patients and KRT devices more readily available. However, because most of the substances that the liver clears are lipophilic and albumin-bound, they are not cleared effectively by KRT. Hence, there has been much effort in developing devices that more closely resemble the clearance function of the liver. This article provides a review of various non-biologic extracorporeal liver support devices that can be used to support these patients, and our perspective keeping in mind the needs and unique challenges present in the LMIC of Latin America.

## Introduction

Liver disease is a significant, yet largely neglected public health issue in low- and medium-income countries (LMIC). Reading the scientific literature, one might get the impression that liver diseases are a more prominent issue in high-income countries, yet it is the LMIC that carry the highest burden (1). Indeed, LMIC have a higher prevalence of disease, higher MELD scores at admission, and an increased risk for inpatient and 30-day mortality. The high prevalence is not only due to the high levels of alcohol consumption (and consequently alcoholic liver disease), but to this one must add the higher prevalence of infectious and toxic etiologies present in many of these regions (2). The worse outcomes are likely related to suboptimal availability of adequate resources, particularly to support patients progressing to liver failure.

Liver failure arises when the loss of functional liver mass approaches critical levels and can no longer maintain its basic functions, including detoxification, biotransformation, excretion, and secretion of numerous endogenous and exogenous substances. While its presentation is variable, it usually includes jaundice, coagulopathy, hepatic encephalopathy (HE), enhanced susceptibility to infections, hemodynamic instability and progressive multiorgan failure and death. Liver failure can be differentiated into acute liver failure (ALF), chronic liver failure, or acute-on-chronic liver failure (ACLF). ALF is when there is a rapid loss of hepatic function in a patient with a previously healthy liver (2, 3). The etiologies vary by region, but include infections (mainly acute viral hepatitis), toxins or drugs (e.g. acetaminophen), cardiovascular failure, or metabolic abnormalities (2, 4–6). It may also develop in patients with multi-organ failure due to severe sepsis (6, 7). The incidence of ALF in Latin America varies between 1 to 8 cases per million inhabitants, peaks between 35-45 years of age, with women accounting for 60% of the cases (8); and accounts for 6% of deaths caused by liver diseases. Acetaminophen-induced ALF is the most common cause, and death ensues due to brain edema/encephalopathy, sepsis, and multiorgan dysfunction (8).

ACLF and progression of CLF are much more common than ALF and typically occur in patients with cirrhosis (9). ACLF is characterized by acute deterioration of a patient with cirrhosis with or without a recognized precipitating event and is associated with progressive multi-organ failure and a high mortality rate (6, 7). Decompensated cirrhosis is pathophysiologically different and typically represents patients who have end-stage cirrhosis with varying degrees of end-organ dysfunction. The most common etiology of these entities in most LMIC (where data is available) are more similar to that of higher income countries; that is, alcoholic cirrhosis is the most common etiology, however with a higher prevalence of other etiologies (e.g. obesity, non-alcoholic liver disease, hepatitis B or C infection, autoimmune disease among others) (10).

Regardless of whether the liver failure is due to ALF, ACLF, or progressive CLF, in its most severe state, liver failure is a medical emergency, that requires supportive care until either the liver recovers, the patient stabilizes, or a liver transplant is performed. Liver transplantation is the ideal therapy, but is not widely available. For instance, in Mexico, despite a 6% annual growth in the number of liver transplants, only until November of 2022 238 liver transplants were performed and there are still 244 patients currently listed for a liver graft in the Mexican registry of the National Transplantation Center (11). This is in the setting of an estimated 350 a 450 patient per year with liver failure that may be eligible for transplant. Thus, strategies to support the patient until the liver recovers or until a liver transplant becomes available are necessary. One such strategy is *via* the use of extracorporeal liver support (ECLS) devices. In order for an ECLS device to replace a normal liver, it should be able to perform the three main liver functions; detoxification, regulation and synthesis. Developing such a device has proved elusive and will likely require utilizing functional hepatocytes (*i.e.* biological devices), which are only available in the research setting. On the other hand, intermediate levels of liver support can be achieved by using currently available extracorporeal blood purification devices. This review will therefore focus on the non-biological extracorporeal blood purification strategies used during decompensated liver disease and failure, emphasizing the more impactful issues in LMIC and in particular among Latin American countries.

## Kidney replacement therapies

The most frequently used type of extracorporeal blood purification used in liver disease remain the different modalities of kidney replacement therapies (KRT). They are, of course, predominantly used for those patients who have liver failure together with concomitant acute kidney injury (AKI), which together with other supportive therapies, can help optimize the patient for liver transplant. While intermittent hemodialysis (IHD) and continuous kidney replacement therapies (CKRT) can be used, CKRT is usually preferable in the critically ill because; a) it does not compromise hemodynamic stability as much, b) offers better control of volume status and c) does not cause rapid fluxes of fluid and electrolytes, in turn decreasing the risk of disequilibrium syndrome and their complications. Moreover, they can be safely used to slowly correct hyponatremia in oliguric patients, thereby decreasing the risk of osmotic demyelination syndromes; a complication to which this patient population is very susceptible. Finally, since, ammonia has a similar kinetic profile to urea (12), KRTs are quite effective at its removal. Indeed, it is used to decrease ammonia levels in infants with disorders of the urea cycle. Hence, some authors have extended the classic indications for CKRT in patients with decompensated cirrhosis to include those that have ammonia levels >200 µmol/L, or 150 µmol/L together with encephalopathy. The KRT is used in conjunction with a bowel regimen (lactulose, lactitol, etc.), until the arterial ammonia level drops below 100 µmol/L (13).

Attempts to clear a larger variety of toxins more efficiently, led to the development of variations of CKRT that utilized higher rates of hemofiltration, which *via* the increased convective clearance would in theory lead to greater clearance of middle size molecules, and consequently ameliorate symptoms and major complications, such as cerebral edema attributed to hepatic encephalopathy (12). One such modality which has been attempted is Sequential High-Volume Hemofiltration. However, the results have been disappointing and the modality has not improved outcomes. Moreover, it has several drawbacks. First, while the higher rates of clearance achieved with these techniques may improve the metabolic profile, it can lead to a situation analogous to dialysis disequilibrium syndrome because of too rapid clearance, and thus worsen cerebral edema and increase intracranial pressure. Second, the rapid clearance can also cause too rapid a correction rate in patients with hyponatremia, particularly if hypotonic fluids are not given concurrently at appropriate rates. Third, they can accelerate amino acid loss, thus producing a negative nitrogen balance (14) which may be particularly detrimental to patients with cirrhosis, particularly in ACLF, as they frequently are protein malnourished (15). Thus, the nutritional requirements of these patients must be carefully evaluated and dietary adjustments made that account for the KRT-associated negative nitrogen balance (14).

**Figure 1:**
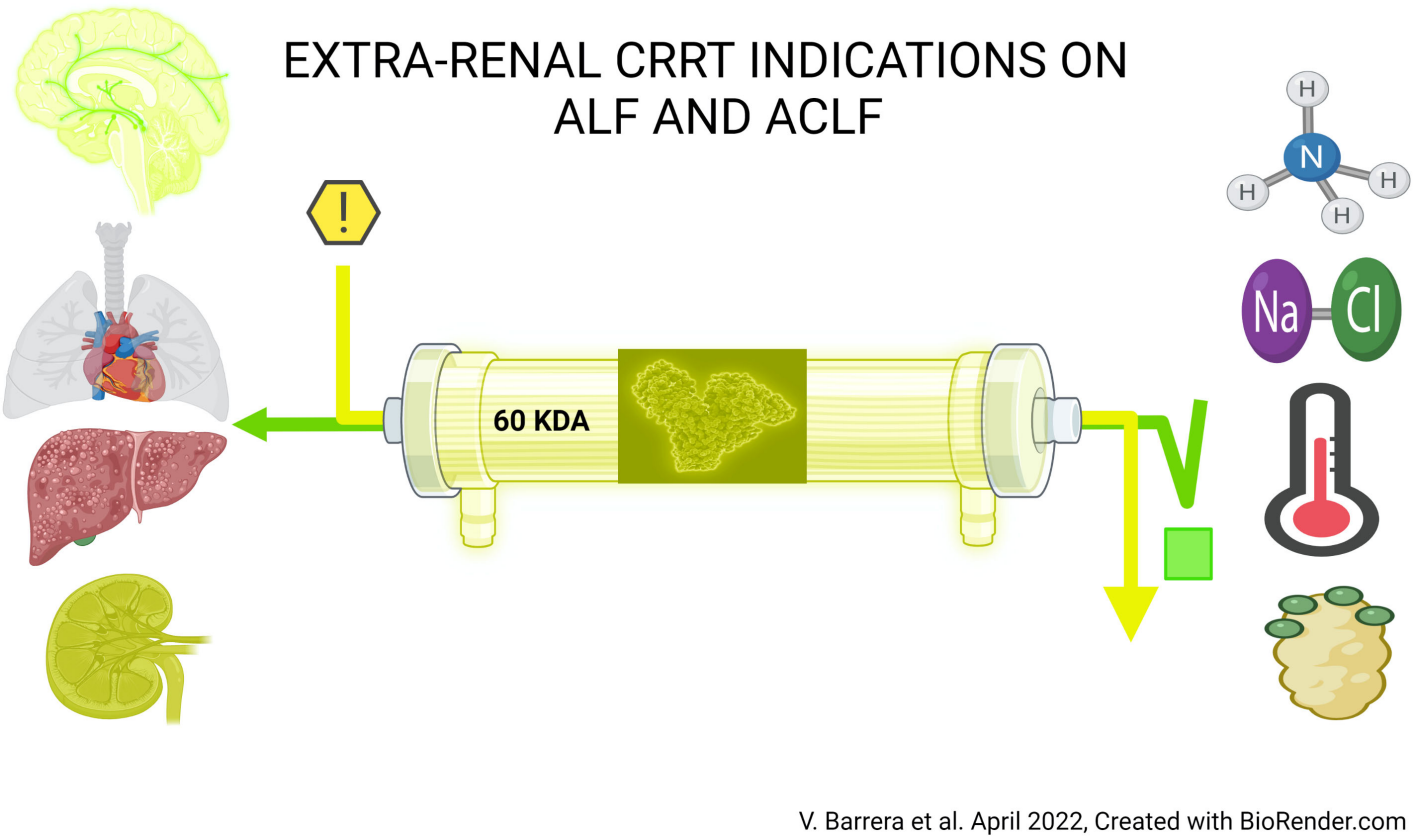
Extra-renal CRRT indications on ALF and ACLF.

While, all the modalities of KRTs play a role in the treatment of patients with compensated and decompensated liver disease with renal dysfunction, they are restricted to removing small, water-soluble molecules that are not substantially bound to plasma proteins and thus of limited efficacy in removing the common toxins associated with liver failure, which are predominantly hydrophobic molecules carried by albumin (13),. It therefore comes as no surprise that KRT systems do not improve survival in patients with liver failure (16). Consequently, there has been much effort into developing strategies that more closely resemble the normal liver’s clearance profile; that is, systems that have the ability to remove lipophilic substances (e.g. bile acids, bilirubin, medium-chain fatty acids, aromatic amino acids) and other protein bound substances. Because the predominant plasma protein carrier of these toxins and waste products is albumin, this protein is targeted by two of the purification strategies (total plasma exchange and albumin-based clearance techniques), whereas adsorption membranes are used in the third strategy (**Figure 1**).

## Therapeutic plasma exchange and high-volume TPE

**Figure 2:**
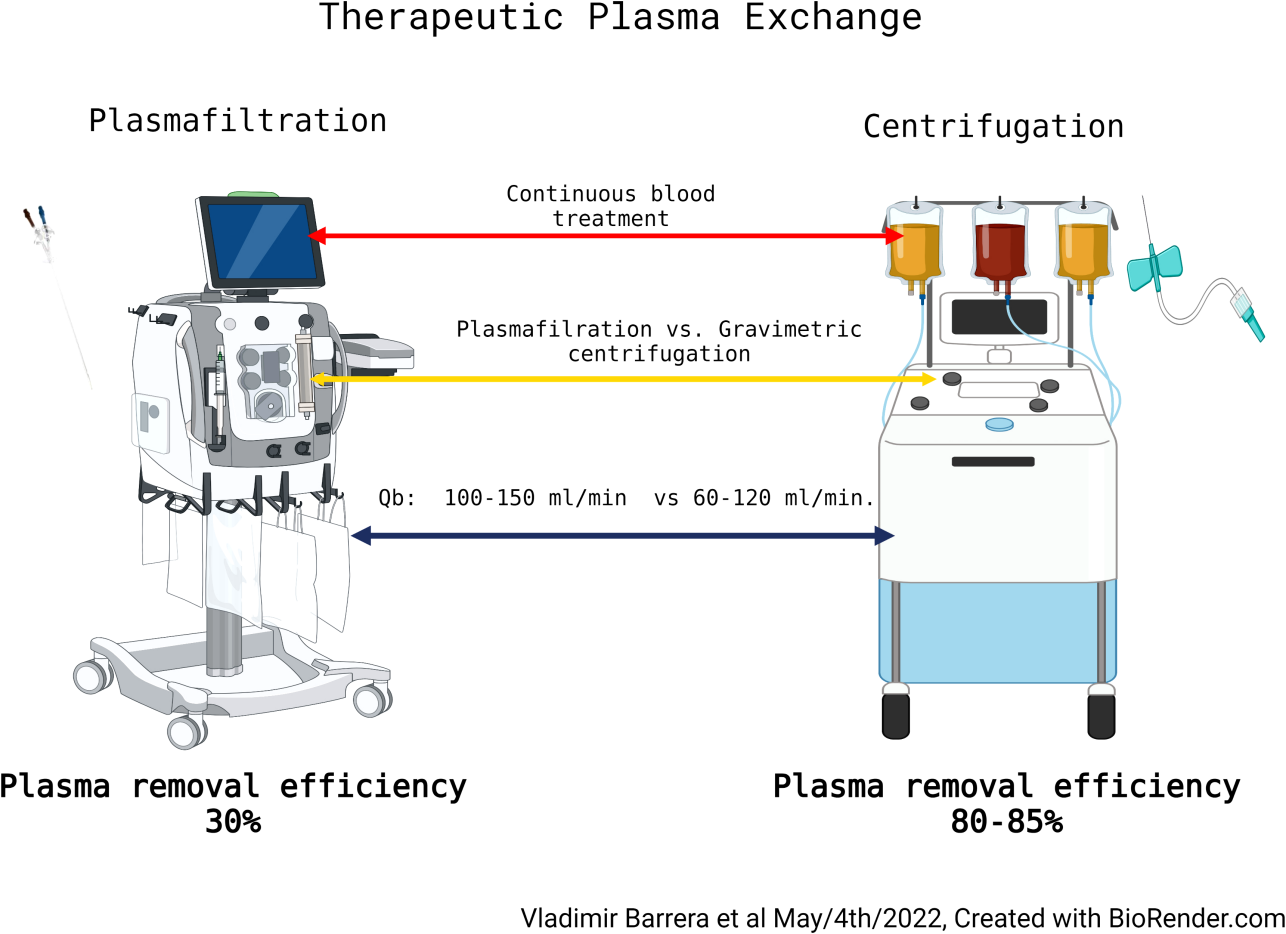
Therapeutic plasma exchange.

TPE has used in liver failure patients since the 1960s. It is the procedure by which the patient’s plasma (containing immune complexes, antibodies, cytokines, endotoxins, and compounds bound to albumin and other proteins) is replaced with a ‘clean’ plasma replacement fluid, predominantly albumin, with varying amounts fresh frozen plasma. The key step in this procedure is the separation of the patient’s plasma from the remaining blood, which can be accomplished by either a) centrifugation, which involves separation by density, or b) filtration through a high cut-off hollow fiber filter that allows the passage of plasma proteins, but not cells (17). Both techniques are frequently used but vary significantly depending on the country. Both have advantages and disadvantages, but filtration-based TPE is gaining in popularity, particularly in limited income countries, due to the simplicity of the devices used. Indeed, filtration TPE is now being performed using standard KRT devices that have been outfitted with specialized plasma filter sets. While this technique is quite efficient, one must be aware of the differences as compared to centrifuge-based TPE, as they differ significantly in their plasma removal efficiency (PRE; the ratio between the removed plasma and processed plasma). Indeed, filtration-based TPE has a significantly lower PRE (30-35%) because of the inefficiencies created from the shorter treatment time, and the increased priming volume used to reduce clotting and clogging of the filter. On the other hand, centrifugal-based TPE can achieve PREs of 80-85% (18). An additional advantage is that, unlike filtration-based TPE, it does not require central venous access. The differences in PRE between the techniques becomes important when calculating the duration of therapy (**Figure 2**

HV-TPE, where one exchanges more than 8-12 liters of plasma (as opposed to 1.5 times the plasma volume), has been proposed as a first-line treatment for ALF of all etiologies, including patients with fulminant ALF (3). The rationale behind this modality is that the large volumes allow for toxin removal from multiple body compartments. Indeed, HV-TPE has been reported to improve metabolic parameters (e.g. it reduced cytokine, endotoxin, and ammonia levels), and clinical parameters, such as encephalopathy, and tissue perfusion (e.g. hepatic, mesenteric and cerebral blood flow), leading the authors to suggest that it can be used as a bridge to transplantation or recovery (4, 9). However, there are limited to no data regarding hard clinical end points, particularly in ACLF, and there are potential complications to the therapy which must be considered. In addition to the usual risks of infection and allergic reactions, hypocalcemia can occur when using citrate as anticoagulation, thrombocytopenia has been reported to occur, particularly with filtration-based TPE. Consequently, while promising, definitive studies establishing its role in liver failure are lacking.

## Albumin based extracorporeal bood purification

Albumin is a 66 KDA multifunctional protein synthesized by the liver (19) with adsorptive and anti-inflammatory properties. Its high net charge, promotes binding of proteins, bilirubin, bile acids, medium-chain fatty acids, and toxins. Indeed, most of the common toxins associated with liver failure are albumin dependent, hydrophobic molecules (20). Hence, detoxification systems that use albumin as a binding substrate allowing for a broader range of toxin removal, compared to standard CKRT were developed (21). There are three main types of procedures based on albumin dialysis, Single-Pass Albumin Dialysis (SPAD), the Molecular Adsorbents Recirculation System (MARS), and Fractionated Plasma Separation and Adsorption (FPSA).

### Single-pass albumin dialysis

**Figure 3:**
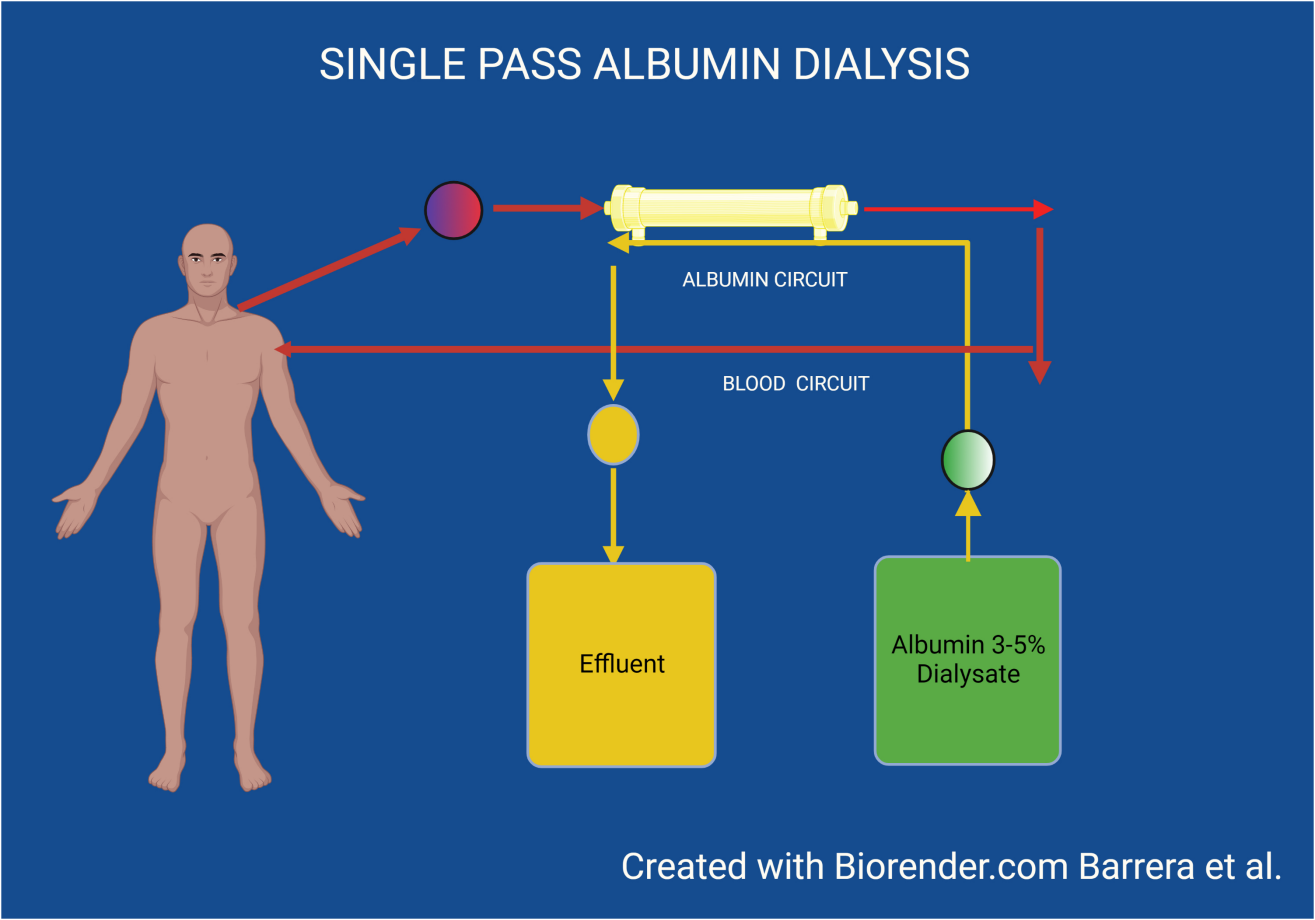
Single pass album dialysis.

Single-pass albumin dialysis (SPAD) is the simplest of the albumin dialysis techniques to implement, as it is performed using standard KRT equipment, making it more readily applicable to many centers. The procedure per se is almost identical to KRT in that the patient’s blood flows through a standard dialyzer or hemofilter, but the dialysate contains albumin, thus enabling the removal of protein-bound molecules that are small enough to pass through the membrane pores. In this system, the dialysate is discarded with the rest of the spent effluent thus it is used once (single pass), thereby necessitating the use of significant quantities of albumin, which constitutes its major shortcoming (**Figure 3**).

While SPAD has been performed using different KRT machines and protocols, the most common modality is *via* CVVHD or CVVHDF using a standard CKRT circuit. The treatment time varies according to the patient’s needs, but is usually in the 6 to 8-hour range, followed by a reassessment of the patient’s status. Albumin is added to standard bicarbonate-based dialysate to a concentration ranging between 1% to 5% (10 to 50 g/l) so that it acts as a molecular adsorber. The addition of albumin to the dialysate should of course be undertaken following strict sterile procedures under a laminar flow hood (21). While alterations in the final electrolyte concentrations will invariably occur, due to the mixing of the solutions, they are rarely of clinical significance, but should be considered as they can impact select patients. Optimal or minimal dialysate flow rates are not established and thus largely established according to local preference, as well as economic and logistical factors. For instance, dialysate usage of 2 l/hr (to achieve ~ 25 ml/kg/hr effluent dose) using an albumin concentration of 3%, results in a consumption of 1440 g/24 hours, this in some centers could be expensive, which can be very expensive (depending on regional costs of albumin), and can also consume large reserves of the hospitals albumin stores, thus limiting its availability for other uses. Consequently, careful planning of albumin use is required. In order to minimize albumin utilization, while maintaining sufficient clearance, many centers opt to decrease the albumin concentration in the dialysate to 1%, and/or using CVVHDF–SPAD with albumin-containing dialysate flows of 1 l/h and completing the overall CKRT dose with non-albumin containing replacement fluids (20). Because treatment times are rather short, as compared to standard CKRT, anticoagulation is often not necessary. However, some centers prefer to routinely administer it to prevent the loss of the circuits or the decreased efficacy of the purification process. When used, the same heparin or citrate protocols used for CKRT are utilized (22, 23).

There is limited evidence of the efficacy of extracorporeal liver support therapy in patient’s’ with liver failure, most of which has been collected in ACLF patients. This is especially true with SPAD where most of the available data is essentially limited to case reports, case series, and small underpowered studies. *In vitro* and *in vivo* studies have shown that it has reasonable efficacy in improving biochemical parameters (e.g. bilirubin and ammonia levels) (24) but the clinical studies have failed to show improvement in hard clinical outcomes. For instance, SPAD did not improve survival or transplant referral in patients with either ALF or ACLF (25, 26). However, the number of patients treated were small, making it impossible to conclude whether it may benefit certain patients. They do suggest that SPAD is effective at improving the biochemical parameters (e.g.) in a safe manner, without adversely effecting hemodynamic or other relevant clinical parameters.

### Molecular adsorbent recirculating system

**Figure 4:**
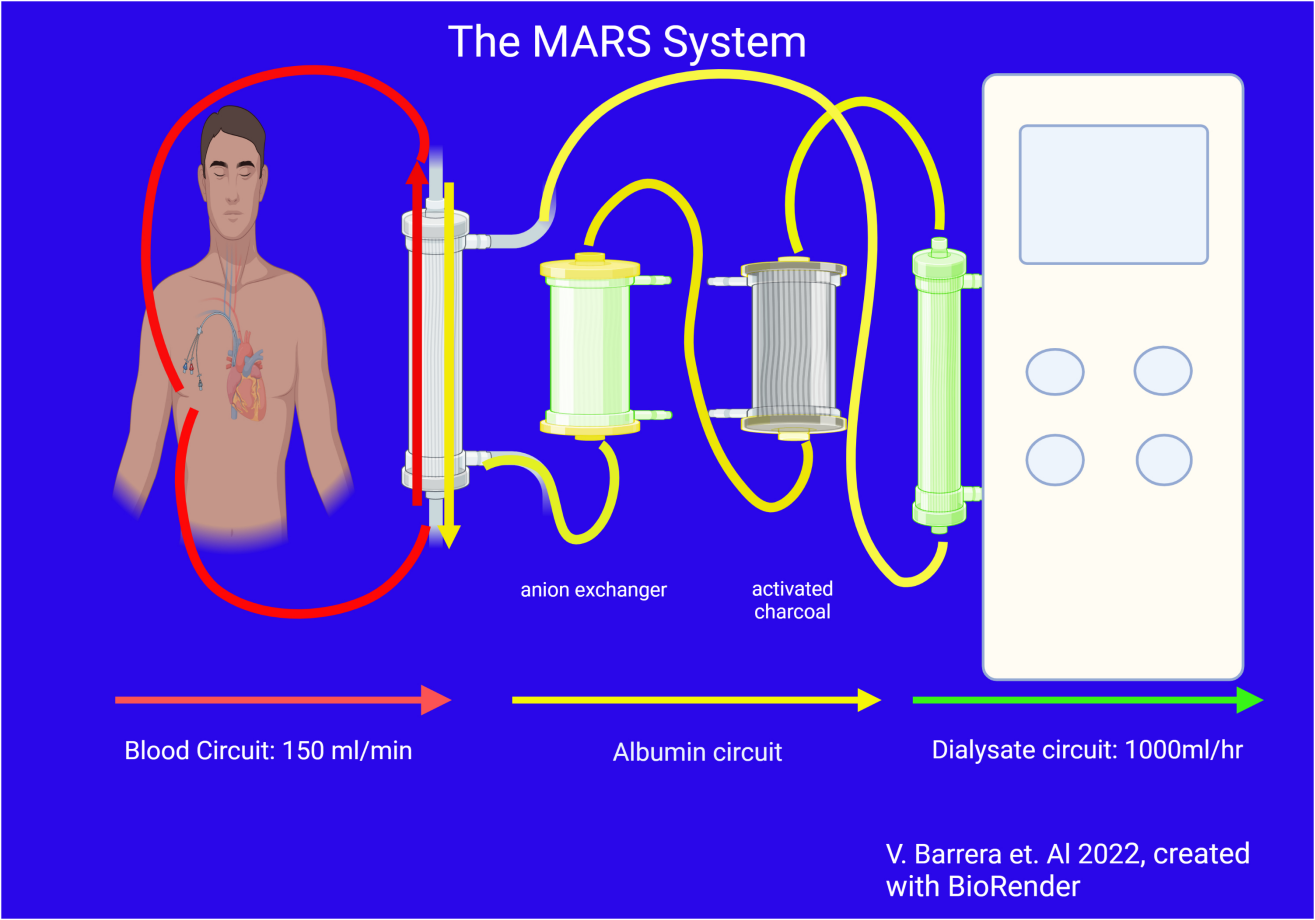
The MARS system.

The MARS system, developed in 1993 by Stange et al., is similar to SPAD in that it also uses a dialysis-like process with albumin as the molecular adsorber. However, in this case, the ‘spent’ albumin is recycled after going through the dialysis filter, rather than being discarded. The MARS detoxification procedure is performed using a specific MARS module monitor running in conjunction with a CKRT device. This system consists of a pair of coupled circuits that act in tandem to detoxify the patient’s blood. In the first circuit, the patient’s blood is dialyzed through a high-flux dialysis membrane (MARS Flux; 50 kDA molecular cutoff) using dialysate containing 20% human albumin. This allows for clearance of small toxins not usually bound to albumin, as well as those that dissociate from the plasma albumin, due to the favorable concentration gradient created by the dialysate albumin (it does not clear substances that bind that are tightly bound to albumin) (27). The spent albumin-enhanced dialysate is subsequently cleansed *via* a multistep process. It is first subjected to dialysis using a high-flux filter to remove water-soluble toxins, followed by the sequential passage of the albumin-dialysate across 2 sequential adsorbent columns containing activated charcoal (diaMARS AC250) and an anion exchange resin (diaMARS IE250). The purified albumin-dialysate is then recirculated back through the first filter, serving as “fresh” dialysate against the patient’s blood. A typical session lasts ~8 h, after which the albumin-regeneration capacity of the adsorbers decreases due to saturation (**Figure 4**).

Applying MARS to patients with ALF and ACLF has been found to consistently reduce bilirubin, bile acids, ammonia, urea, lactate, and creatinine levels (16). Moreover, an improvement in circulating neurohormones, nitric oxide, and oxidative stress, with concomitant improvement in cholestasis, pruritis, liver function, renal function, encephalopathy, and, blood pressure have been reported (28, 29). However, whether these benefits translate to a benefit in clinical outcomes has been less clear. A single-center retrospective cohort study from Mexico suggested that MARS provided sufficient support that allowed the native liver to recover, thus reducing the need for liver transplantation (30). Another retrospective study suggested that MARS may be of value as a bridge to transplant but also revealed severe side effects with respect to coagulation and electrolytes (31). However, retrospective trials are mainly hypothesis generating and must be interpreted with caution. The largest trials available, including the RELIEF and FULMAR (32, 33) trials, did not show a survival benefit, although patient heterogeneity and a short time on therapy may have limited the studies abilities to detect benefits. Regardless, the evidence for efficacy of MARS on hard clinical outcomes is modest. Although it has generally been found to be safe, significant bleeding episodes have been reported. Because patients with liver failure already have a high risk of bleeding, it may be prudent to be vigilant of this complication (34).The comparative efficacy of MARS vs SPAD has been studied by several trials (20). While minor differences in clearance of urea and creatinine (and perhaps bile acids) were suggested, no significant differences in the clearance of the more relevant liver-related biochemical factors, nor of clinical parameters (e.g. encephalopathy, pruritus, and hepatorenal syndrome) were detected. It is important to note that in the U.S. it is only approved for the treatment of intoxications by drugs that are removed by albumin-based dialysis or hemoperfusion.

### Fractionated plasma separation and adsorption

**Figure 5:**
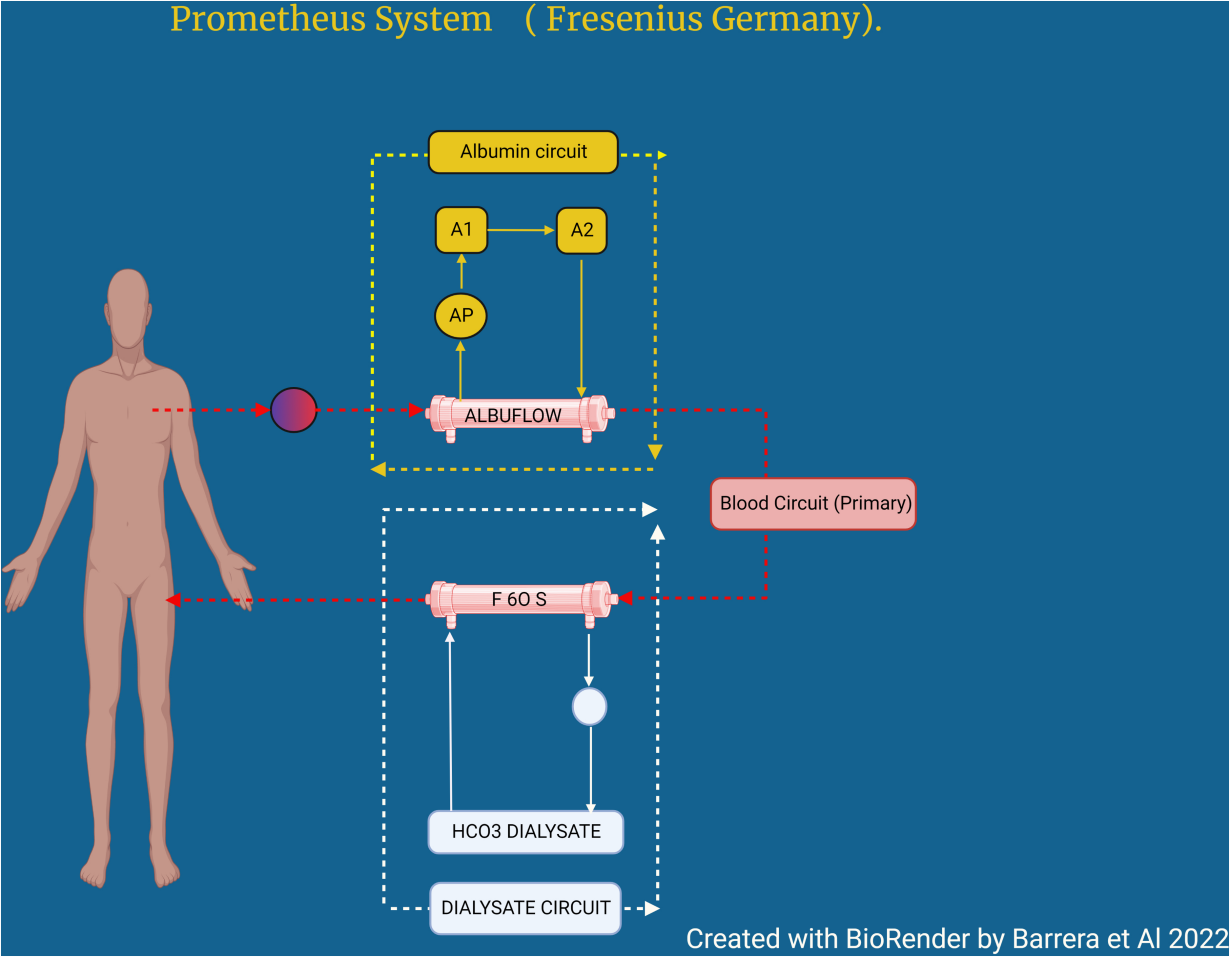
Prometheus system (Fresenius Germany).

The Prometheus FPSA system combines albumin adsorption with high-flux IHD after selective filtration of the albumin fraction through a polysulfone filter. Specifically, the patient’s blood first passes through an albumin-permeable biocompatible filter (Albuflow^®^; molecular weight cutoff of 250kDa), filtering out an albumin rich plasma fraction which then passes through a neutral resin adsorber (Prometh^®^ 01) and an anion-exchanger (Prometh^®^ 02) before being returned to the blood. The reconstituted blood then undergoes conventional dialysis using a high-flux polysulfone dialyzer. As with MARS, the saturation of the adsorber columns cause a decline in toxin clearance rates after >6 h (**Figure 5**).

As with SPAD and MARS, use of the Prometheus FPSA can improve metabolic parameters (e.g. bilirubin, ammonia, lactate, urea, and creatinine), as well as various clinical parameters (e.g. pruritis and hepatic encephalopathy). Despite these advantages in biochemical parameters, the clinical benefits are less consistent. While several studies have found improvement in certain clinical parameters (e.g. encephalopathy, pruritis, renal function), these have not been consistent and most have not found an improvement in outcomes (35, 36). Indeed, the largest randomized controlled trial failed to find a survival benefit (37). Whether a specific subset of patients may benefit (38), remains unproven.

## Adsorption membranes and columns

Because liver failure, particularly ACLF is associated with an inflammatory response, there has been much interest in developing techniques that could remove the mediators driving this response (e.g. cytokines). The failure of traditional CKRT techniques to provide clearance of cytokines, has led to the resurgence in hemoperfusion techniques. Hemoperfusion is the method by which blood is cleared of various compounds by directly perfusing it over a sorbent bed or column, which have a binding affinity for the substances to be removed. The sorbent used may be activated charcoal, non-ionic or ionic resins, or immunosorbents. Adsorption of the solute to the sorbent is based on chemical affinity, rather than molecular size. While early sorbents had significant safety concerns, the development of more biocompatible materials have resulted in increased safety and a range of sorbents are being tried in various clinical settings including liver failure. Indeed, they are an integral part of the MARS and Prometheus circuits described in the previous section. But newer sorbent cartridges raise the possibility of being able to obviate the use of albumin by directly perfusing the blood through these cartridges. Examples of such cartridges being tested in liver failure include:

- Cytosorb is a cartridge with 300ml container filled with a biocompatible, highly porous polymer bead designed of polystyrene, a divinylbenzene co-polymer that can absorb medium molecular weight molecules, including albumin-bound toxins like indirect bilirubin and bile acids, even faster than MARS (39).
- A double plasma molecular adsorption system that incorporates two cartridges in tandem, the HA 330 II (a neutral microporous resin) and BS 330 (an anion exchange resin) (40). This setup not only removes bilirubin, bile acids and ammonia (with comparable efficacy to TPE), but also removes inflammatory mediators (e.g. Il-6, TNF-a) thus modulating the inflammatory response. It has been used to treat the complications of liver failure like hepatic encephalopathy, jaundice (41).

Despite their promise, evidence of efficacy is even more scarce than with albumin-based blood purification techniques. Thus, their use should mainly be under the auspices of a study or for compassionate use in select cases, pending further studies being completed.

## Considerations when choosing a therapy in LMIC

When implementing a complex therapy (e.g. ECLS) into a healthcare system, particularly in regions with limited resources, one must consider several issues including, a) efficacy of therapy, b) equipment, supplies and disposables, c) personnel-related (training, and maintenance of expertise) d) logistics, e) economics, and f) criteria for patient selection (**Table 1**). Careful consideration into the nuances of each of these issues must be given, as they will heavily influence the choices we make.

**Table 1:** Comparative of technical aspects between extracorporeal liver assist devices.

		CKRT	TPE	SPAD	MARS	FPSA	Adsorption Filters
Availability		++	+	++	+/-	+/-	+/-
Clearance	Small solutes	+++	+	+++	+++	+++	V
	Midsize solutes	+/-	+	+/-	+/-	+/-	V
	Large solutes	-	+	-	-	-	V
	Albumin Bound solutes	-	++	+	+	++	V
Albumin Usage		–	+++	+++	++	++	–
Anticoagulation		+/-	+	+/-	+/-	+/-	+/-
Capital Equipment		$	$/$$	$	$$$	$$$	$
Proprietary Disposables		Yes/No	Yes	Yes/No	Yes	Yes	Yes
Training / Maintenance *		1	2	1	3	3	2
Cost		$	$$	$$	$$$	$$$	$$$

* Increasing levels of difficulty of maintenance of expertise.

V, Variable. Depends on filter used.

*CKRT.* As mentioned previously, CKRT has not been shown to provide a clear benefit over IHD when managing patients with AKI, but it has some advantages in treating the sickest patients, especially those with liver failure; it has less of an effect on blood pressure and intracranial pressures. Unfortunately, the availability of CKRT devices in LMIC is much less than in upper income countries, and they are only available in larger centers and cities, if at all. There are numerous barriers to widespread implementation of this technology. The first is the availability and the costs of the devices. For instance, the 2 largest manufacturers of the CKRT devices in North America, do not offer their products to most of the Latin American countries. Moreover, even when machines are available, it can be a challenge to secure appropriate solutions and the proprietary disposables (filters, tube sets, and cartridges). Often times the costs of these supplies severely limit the ability to use the products. This aspect is often compounded by the lack of insurance coverage for these services. Finally, ensuring continuous training of users (e.g. nursing) can be challenging when utilization is low. Despite this, the use of CKRT is increasing in Latin America and may be a useful component in supporting patients with liver failure, particularly those awaiting a transplant.

*TPE.* It is interesting to note that EASL guidelines favor TPE (3), despite the paucity of evidence. Until recently, this would have been difficult to implement in many regions due to the specialized equipment required. However, the development of filtration-based TPE raises the possibility of increasing its implementation. The advantages include being able to perform the therapy with the same dialysis or CKRT machines used for providing KRT (thus decreasing equipment costs), the simplicity of the technique, which makes it easier to train and maintain expertise of the nursing personnel. However, numerous challenges remain. The increased use of blood products (e.g. plasma and albumin) presents new challenges not only in ensuring that adequate supplies are secured, particularly if HV-TPE is being considered, as each session would use ~500 grams of albumin, but also the very substantial cost impact must be considered (discussed in next section). Thus, appropriate logistical considerations including frequent reassessment of product levels must be undertaken to ensure adequate storage levels, as daily needs will vary greatly depending on changing usage patterns. Finally, if the patient is also on CKRT, the interruption of CKRT may increase the number of filters used, representing another source of increased costs (42).

*Albumin based therapies: (SPAD vs. MARS)* Of the albumin-based extracorporeal blood purification strategies, MARS and Prometheus are the most studied. While the Prometheus FPSA system has several theoretical and clearance advantages, it is not available in LMIC. When comparing MARS to SPAD, there is no clear advantage of either system in liver failure. The preference of treatment hence depends upon other factors such as availability, cost and other factors. In this respect, SPAD is likely more applicable in LMIC because it can be performed with standard KRT devices, whereas instituting MARS requires the use of a dedicated CKRT device in conjunction with the MARS device, and thus increasing capitol and maintenance costs. It’s complexity also makes it more challenging to maintain nursing expertise. The main extra cost associated with SPAD is that associated with the increased albumin use. In Mexico, the cost of a 20% albumin bottle varies from site to site. The Mexican Social Security Institute website reported a cost of around $25 US dollars (USDs), although in some drugstores it could be found between $30-40 USD. In other countries like Dominican Republic, the cost goes up around $90 USD per bottle. Thus, though albumin is expensive and increases the cost per dialysis, it still appears to be more sustainable at this time in LMIC, than the other albumin-based modalities.

## Conclusion

Liver failure is an important cause of morbidity, mortality and healthcare costs in LMIC. The limitations of timely transplantation make it essential that we implement the usage of ECLS strategies as a bridge to recovery or transplant in appropriate patients. Applying this in a timely manner may facilitate the transplantation procedure, by optimizing the patient pretransplant, and perhaps decreasing the risk of chronic sequelae following liver transplant (43). These goals can be accomplished in LMIC predominantly *via* KRT/CKRT, TPE and perhaps SPAD.

## Author contributions

VB had the original idea. VB, LR-T, LJ corrected and arranged the bibliography and information. VB, LR-T, LJ made the review design and wrote the text. VB and LJ designed the figures. LR-T, LJ, LB-F, EC, DB and OS reviewed the final text and the figures. LR-T and LJ contributed equally to the study as senior authors. All authors contributed to the article and approved the submitted version.
